# Spontaneous Agglomeration of Fluorinated Janus Particles and Its Effect on the Adsorption Behavior of Oil-Air Surfaces

**DOI:** 10.3389/fchem.2020.602424

**Published:** 2021-01-08

**Authors:** Gen Li, Keliang Wang, Chunjing Lu

**Affiliations:** ^1^Department of Petroleum Engineering, Northeast Petroleum University, Daqing, China; ^2^Key Laboratory of Enhanced Oil Recovery, Northeast Petroleum University, Ministry of Education, Daqing, China

**Keywords:** pickering emulsion, Janus particle, oil foam, particle agglomerate, Cassie–Baxter

## Abstract

Based on the Pickering emulsion template method, two types of Janus particles with different relative amphiphilic areas for stabilizing non-aqueous foam were synthesized. In addition, particles with uniformly modified surface were synthesized for comparison. By adjusting oil mixtures, the behavior of particles on the oil-air surface was measured. Moreover, the role of particle agglomerates in surface adsorption process was investigated. Affected by the particle surface contact angle, the surface activity of Janus particles is not always greater than that of uniformly modified particles, which is reflected on delta surface tension and the volume of foam generated. The oil-surface adsorption process of synthesized Janus particles is not only occurred in the form of independent detached particles, but also in the form of particle agglomerates. The adsorption of the particles from the bulk phase to the surface requires the contact angle of the Cassie–Baxter composite surface of the particle agglomerates to be around 90°, but the inherent contact angle of the individual particles is <90°.

## Introduction

In the past two decades, foams stabilized by colloidal particles have attracted the interest of many researchers (Murray and Ettelaie, 2004; Sun et al., 2015; Asghari et al., 2016; Narsimhan, 2016; Wang and Nguyen, 2016; Sakthivel et al., 2019). Most of aqueous foams with water have been investigated extensively and intensively (Sheludko, 1967; Pugh, 1996; Georgieva et al., 2009; Bournival et al., 2015; Davarpanah et al., 2019; Emadi et al., 2019; Ahmed et al., 2020; Singh and Mohanty, 2020). But non-aqueous foams, such as air bubbles dispersed in oil, have not been sufficiently studied. However, these non-aqueous foams play an important part in cosmetics, food, medicine, petroleum, and manufacturing. In particular, these foams can be invoked as makeup removers, drilling fluids, fracturing fluids, and solvent-based cleaners.

In particle-stabilized foam, an irreversible particle adsorption layer on the bubble surface can act as a physical barrier against coalescence and disproportion (Binks and Horozov, 2005; Kaptay, 2006; Horozov, 2008; Hunter et al., 2008; Hussain et al., 2019). The effective adsorption of particles on surfaces is a prerequisite for achieving the stabilization function of colloidal particles. For solid-liquid-air systems, if the sum of solid-air tension γ_sa_ and solid-liquid tension γ_sl_ is less than the original liquid-air tension γ_la_, the adsorption process is thermodynamically advantageous. Some studies (Binks and Fletcher, 2001; Binks and Rocher, 2010; Binks et al., 2011; Binks and Tyowua, 2013) have suggested that the adsorption of particles on the liquid-air surface is significantly related to the wettability of the particles, which is quantified by the contact angle θ between the particles and the liquid-air surface. The contact angle is given by Young's equation:

(1) $$\begin{array}{l} \text{cos}\theta =\frac{\gamma_{\text{sa}}-\gamma_{\text{sl}}}{\gamma_{\text{la}}} \end{array}$$

When the liquid phase is oil, the particles must be partially oleophobic, that is, θ is between 0° and 180°. According to calculations, when θ = 90°, particles are most easily adsorbed on the surface (Binks and Fletcher, 2001; Alargova et al., 2004; Dickinson et al., 2004). This implies that the particle surface must have some repellency to the oil to obtain intermediate wettability.

In general, the high surface tension caused by hydrogen bonds at the water-air surface makes the aqueous surface extremely susceptible to adsorption of surface-active components, and such aqueous systems are prone to foaming. However, other fluids, such as hydrocarbons, have significantly lower surface tension. In addition, the driving force for the surface of these fluids to be adsorbed by surface-active substances is relatively small, which leads to a significant decrease in foamability and stability. One of the focuses of oil foam research is to ensure that the particles are adsorbed on the oil-air surface. Since the surface energy of fluorocarbons is lower than that of hydrocarbons, fluorocarbons can be utilized to reduce the surface tension of oil. Studies have shown that fluorocarbon-based surface-active substances can be adsorbed onto the surface and reduce the surface tension of hydrocarbon oils, thereby stabilizing the oil foams (Binks and Fletcher, 2001; Shrestha et al., 2006, 2008, 2010; Binks et al., 2010, 2011; Murakami and Bismarck, 2010).

However, it is difficult for the particles to have an oil-repellent effect simply by controlling the surface chemical properties of the particles. For example, even on smooth polytetrafluoroethylene, the contact angle of liquid alkane is much <90° (Fox and Zisman, 1950). In previous studies, changing the surface roughness was used to enhance oil repellency (Marmur, 2008; Yan et al., 2011; Song et al., 2017; Domingues et al., 2018). This principle is described by the Cassie–Baxter equation (Cassie and Baxter, 1944):

(2) $$\begin{array}{l} \text{cos}\theta_{\text{CB}}=f_{\text{C}}\text{cos}\theta +f_{\text{C}}-1 \end{array}$$

Where *f*_c_ is the solid-liquid contact area fraction. Increasing the value of *f*_c_ can cause oil repellency on the surface of the oleophilic material. Therefore, it is a promising research direction to study oil foam from the surface roughness.

Compared with the homogeneous particles, amphiphilic Janus particles (De Gennes, 1992) can increase the surface activity up to 3 times (Binks and Fletcher, 2001; Glaser et al., 2006). Such particles, which are more easily adsorbed toward the interface, can provide better long-term stability of emulsions and foams. A theoretical study indicates that the free energy of Pickering emulsions stabilized by Janus particles is negative, which is a thermodynamically stable system (Aveyard, 2012). Despite the remarkable properties of Janus particles, the application of Janus particles to oil foams has so far been insufficiently reported.

In this paper, two types of Janus particles with different relative amphiphilic areas were synthesized based on the Pickering emulsion template method. In addition, particles with uniformly modified surfaces were synthesized for comparison with Janus particles. The oil-air surface properties were adjusted by changing the ratio of each substance in the oil mixture, and the behavior of the particles on various oil-air surfaces was measured. In this process we found that particle agglomerates play a significant role in particle adsorption to the surface.

## Materials and Methods

### Materials

Hydrophilic silica particles were purchased from NPS Chemical Company (d50 = 195 nm, specific surface area 14 m^2^/g). 3-aminopropyltriethoxysilane(APS), N-octyltriethoxysilane (OTS), 1H, 1H, 2H, 2H-perfluorodecyltriethoxysilane (PFTS), perfluorooctanoic acid (PFOA), fluorescein5(6)-isothiocyanate (FITC), n-octanoic acid (OA), and triethylamine were purchased from Aldrich. Phosphate Buffered Saline (PBS, pH 7.4) was purchased from Thermo Fisher Scientific. Tricresyl phosphate and n-octane were purchased from Sinopharm Chemical Reagent Company. All products have been used as received.

### Asymmetric Modification by APS

Pickering emulsions were prepared in a similar way to Granick and co-workers (Hong et al., 2006). At 80°C, 0.8 g of SiO_2_ particles was dispersed in 12 g of wax, and then 60 mL of deionized water was added. Pickering emulsion was prepared by stirring at 7,200 r/min for 10 min. Immediately after stirring, the emulsion was cooled in cold water to solidify the wax. Rinse the solidified emulsion droplets with deionized water to remove unabsorbed SiO_2_ particles. Next, the emulsion droplets were chemisorbed with APS (2 × 10^−3^ mol/L) in a methanol solution at 18°C for 25 min. Triethylamine (1.5 × 10^−4^ mol/L) was also used as a catalyst simultaneously. The wax droplets were then washed with methanol to remove excess APS followed by dissolved in chloroform. And the modified SiO_2_ particles were collected by centrifugation and washing. Farther, the particles were annealed at 110°C for 2 h.

### Synthesis of Janus Particles

The SiO_2_ particles partially coated by APS on the surface were dispersed into 80 mL of toluene using ultrasound. And OTS (2 × 10^−3^ mol/L) was added to react at 18°C for 6 h. The particles were re-dispersed in toluene and centrifuged through multiple rounds to remove unreacted OTS.

The silica particles modified with APS and OTS were dispersed in PFOA solution (2.4 × 10^−2^ mol/L) and stirred for 48 h. Then, after removing the solvent by evaporation, the remaining particles were annealed at 110°C for 2 h. These particles were re-dispersed in chloroform and centrifuged over multiple rounds to remove ungrafted PFOA. For ease of expression, we named this particle as JP1.

We used a similar method to make another Janus particle with a different fluorocarbon coverage area. Partially APS-modified SiO_2_ particles were dispersed into 80 mL of toluene using ultrasound. Then, PFTS (2 × 10^−3^ mol/L) was added and reacted at 18°C for 6 h. Unreacted PFTS was removed by multiple rounds of redispersion and centrifugation.

**Figure 1:**
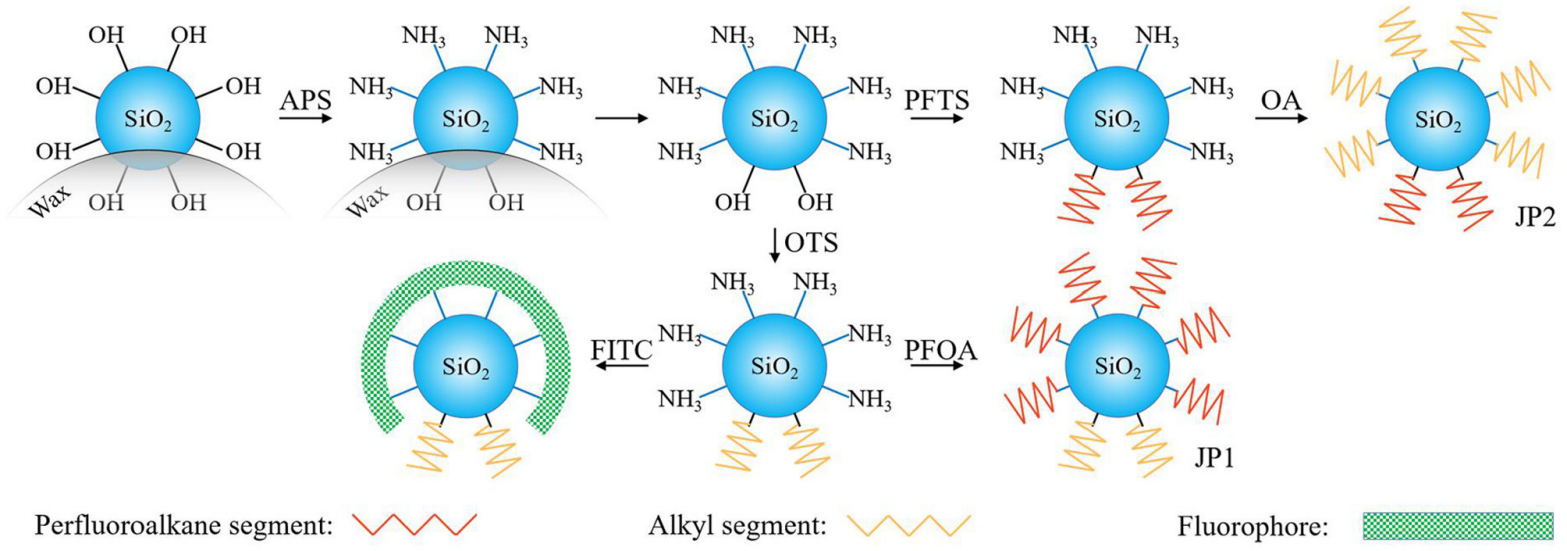
Scheme for the synthesis of two Janus particles from a Pickering emulsion. The exposed and non-exposed areas of silica particles on the wax droplets were inconsistent. No wax covered surface of silica particles is coated by APS and provides a reaction site for further grafting of fluorocarbon chains or hydrocarbon chains.

Next, these particles were dispersed in an OA solution (2.4 × 10^−2^ mol/L) and stirred for 48 h, and then the reaction was terminated by removing the solvent by evaporation. After the particles were annealed at 110°C for 2 h, the ungrafted OA was discarded by centrifugation and washing. We call this particle JP2 and use it later. The above process is illustrated by Figure 1.

### Preparation of Uniformly Modified Particles

Uniformly modified particles with only fluorocarbon chains on the surface were made for comparison with Janus particles. 0.5 g of bare SiO_2_ particles was dispersed into 100 mL of toluene using ultrasound, and PFTS (2 × 10^−3^ mol/L) was added to react at 18°C for 6 h. particles were washed multiple rounds in toluene followed by drying at 110°C.

### FTIR and XPS Measurement

FTIR spectra were taken with a 1615 FTIR (PerkinElmer, USA) spectrometer using KBr pellet pressing method for all bare and modified silica particles. An AXIS-ULTRA DLD X-ray photoelectron spectrometer (Shimadzu, Japan) with a monochromatic Al Ko X-ray source (hv = 1,486.6eV) was used for X-ray photoelectron spectroscopy (XPS) measurement. During the measurement, the incident angle was 90°. The binding energy was corrected with the C_1s_ peak (284.6 eV).

### Optical Microscopy, SEM, and EDS Measurement

The shape and droplet size of Pickering emulsions were observed through an IX73 optical microscope (Olympus, Japan). Morphology of SiO_2_ particles on the surface of wax emulsion droplets was characterized by Sirion200 SEM (FEI, Netherlands). The droplets used for SEM observation were first frozen by liquid nitrogen and sprayed with gold on the surface. Energy dispersive spectroscopy (EDS) analysis of the samples was performed with the SEM equipped with an EDS analyzer.

### Preparation and Observation of Fluorescent Particles

Particles that have been covered with hydrocarbon chains and amino groups were made into fluorescent particles (Figure 1). An acetone/water mixture (1/9, v/v) containing 10 mg of FITC was added to 20 mL of PBS (pH 7.4) in which 0.02 g of APS and OTS coated SiO_2_ particles were dispersed, and the mixture was stirred at 20°C in the dark 12 h. The reaction was stopped by multiple centrifugation and washing with PBS. Finally, the fluorescent particles were observed by a FV1000 laser confocal microscope (Olympus, Japan) at 5°C.

### Preparation of Oil Mixture

In the characterization of the oil-air surface properties of the particles, we obtained various surface tension oil mixtures by adjusting the relative ratio of n-octane and tricresol phosphate. The surface tensions of various oil mixtures was measured by the pendant drop method using a Tracker (Teclis, France). The results are given in Table 1.

**Table 1:** Surface tension of various oil mixtures used for testing at 20°C.

**N-octane: Tricresol phosphate**	**Surface tension (mN/m)**
20:0	21.8
19:1	22.1
18:2	22.6
17:3	23.0
16:4	23.3
15:5	23.7
14:6	24.2
13:7	24.8
12:8	25.5
11:9	26.0
10:10	26.7
9:11	27.7
8:12	28.6
7:13	29.7
6:14	30.7
5:15	31.9
4:16	33.1
3:17	34.5
2:18	36.3
1:19	38.2
0:20	40.9

### Contact Angle Test for Smooth and Rough Solid Surfaces

Instead of using particles, quartz slides coated with fluorocarbon chains were used to measure the intrinsic contact angle of the particle surface. A 3 × 3 cm quartz slide was treated with a piranha solution, and then repeatedly washed with deionized water followed by drying. The pre-treated quartz slide was immersed in a toluene solution of PFTS (6 × 10^−3^ mol/L) and reacted at 18°C for 6 h. After the reaction, the slide was quickly taken out, and then immersed in toluene and chloroform for ultrasonic washing. Finally, the quartz slide was dried at 110°C. A Tracker (Teclis, France) was used to measure the contact angle of a 0.10 mL droplet in the air on the smooth surface of the modified quartz slide.

The modified quartz slide with a spin-coated particle layer was used to represent the rough surface of the particle agglomerates to characterize the contact angle. A SK250H ultrasonic instrument (KUDOS, China) with cold water circulation function was used to disperse 5 wt% particles in cyclohexane. 0.8 ml of the dispersion was placed on the modified quartz slide and coated at 800 rpm for 40 s using a WS-650Hz-8NP/UD3 (Laurell, USA). The rough surface contact angles on these surfaces were measured using Tracker. Three separate measurements were repeated for each oil mixture.

### Wettability and Foamability of Particles

To test the wettability of particles, 0.20 g of particles was poured on top of 3.0 ml of oil in a glass bottle (capacity: 15 ml). Gently shake the bottle from side to side for 10 s and monitor whether the particles are immersed in the oil. Further foaming test was performed by manually shaking the bottle up and down for 15 s at 4 Hz, and then the foam volume was recorded. Each experiment was carried out three times.

### Particle Agglomerate Size and Surface Tension

The particle's oil-air surface tension and agglomerate size were measured under two conditions. One condition is to shake the particles and oil gently for 10 s and take the clear solution as a sample. Another condition is that the particles are dispersed into the oil using ultrasound. Surface tension and the size of particle agglomerates in the oil were measured using a Tracker (Teclis, France) and a Nano ZS (Malvern, UK), respectively. Each experiment was performed three times.

## Results and Discussion

### Characterization of Pickering Emulsions

**Figure 2:**
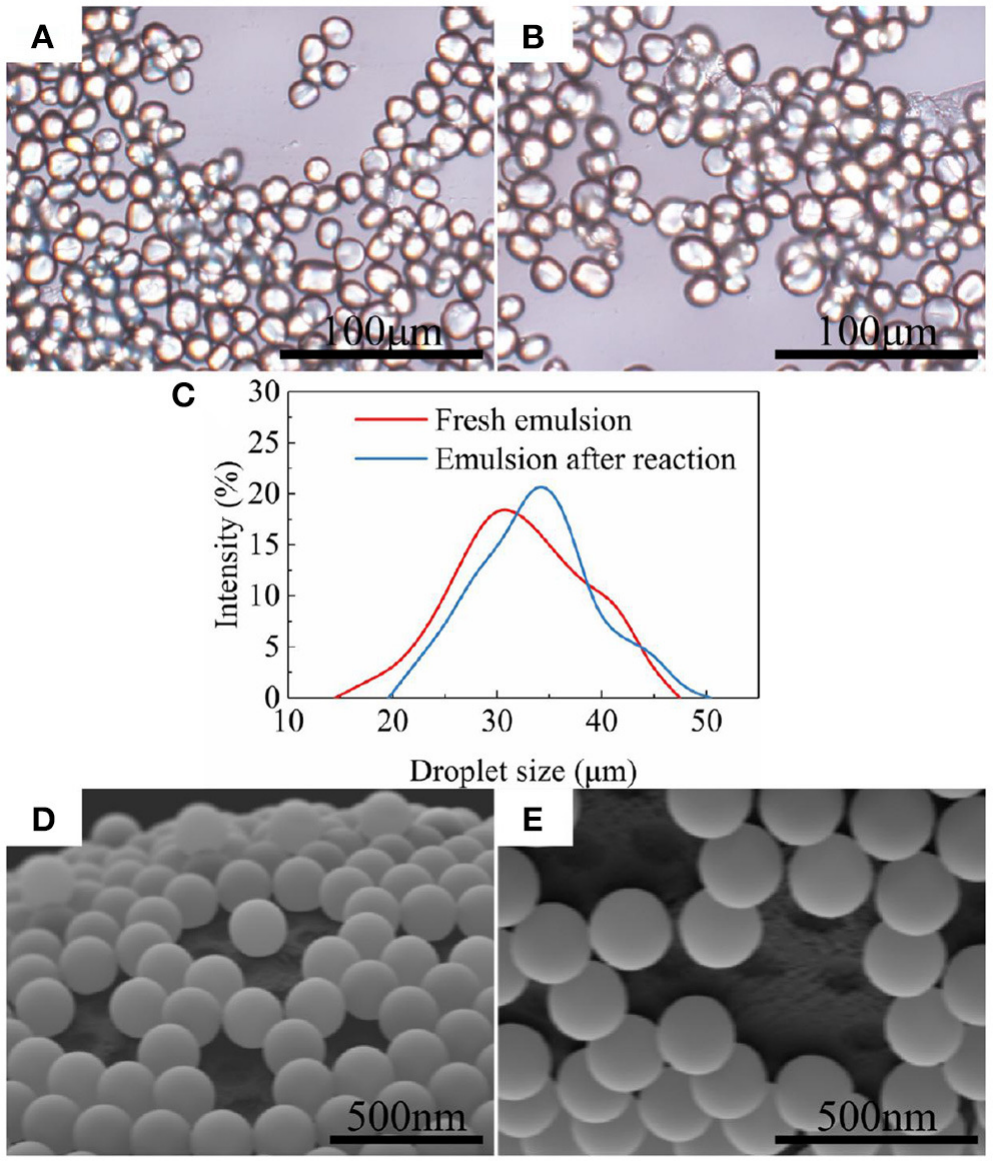
SiO_2_ particles and pits on Pickering emulsion droplets. **(A)** Optical micrograph of the emulsion before APS modified; **(B)** Optical micrograph of the emulsion after APS modified; **(C)** Size distribution of wax droplets before and after modification. **(D)** SEM image of the emulsion before APS modified; **(E)** SEM image of the emulsion after APS modified.

The asymmetric masking of particles by wax is an important initial step in the preparation of Janus particles. The appearance and droplets size of Pickering emulsions are shown in Figure 2. The solidified wax droplets remaining the shape of non-spherical were observed in Figures 2A,B. But wax droplets were slightly swelled by reaction solvent after APS modification (Figure 2C). Moreover, it was found in Figure 2B that some particles fall off from the wax droplets surface and form flocs after the reaction. These exfoliated particles were not well-anchored to the surface of the wax droplets due to their strong hydrophilicity. However, there were still enough particles anchored on the emulsion droplets after the reaction according to Figures 2D,E. Before and after the chemical reaction, the particles on the surface of wax droplets were partially desorbed and left some pits on wax. The surface of the particles masked by such pits is relatively small. Due to the hydrophilic nature of bare silica particles, the adsorption equilibrium particles on the emulsion droplets are exposed to most of surface that provides reaction sites. This inspired us to use only this Pickering emulsion to prepare two types of Janus particles with different relative coating ratios of fluorocarbon and hydrocarbon.

### Analysis of Chemical Bonds on Particles

**Figure 3:**
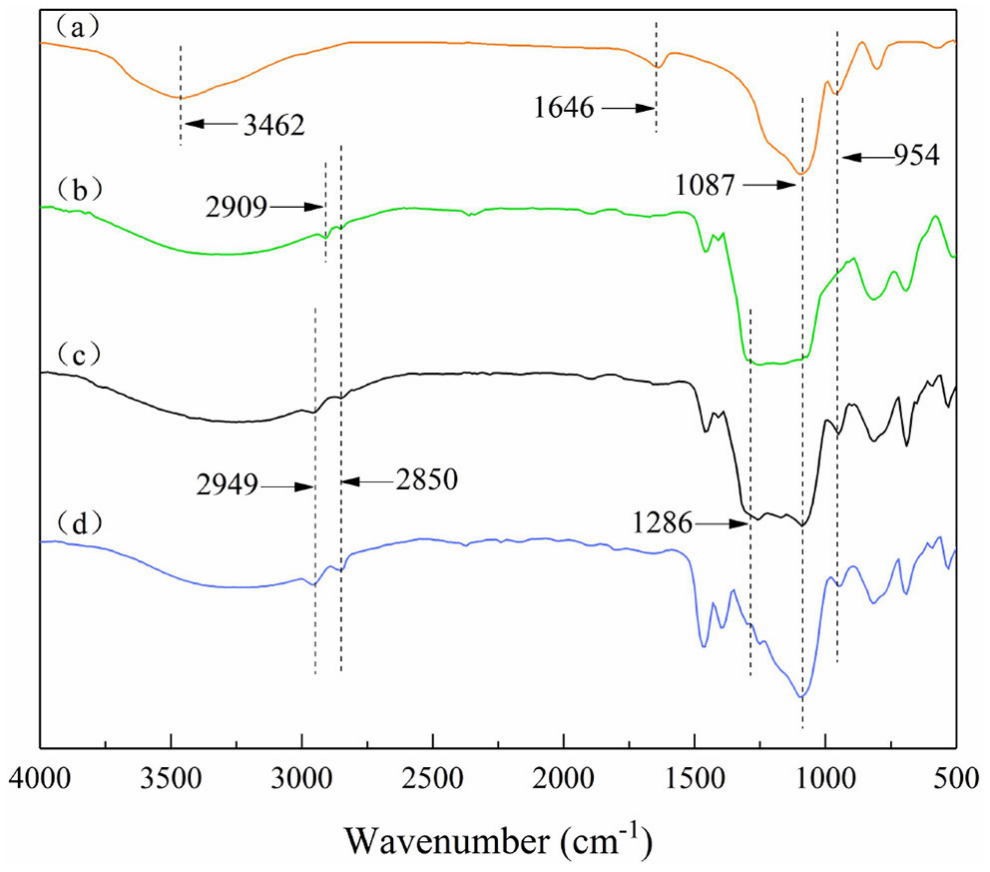
Fourier transform infrared spectra of particles. **(a)** Bare silica particles; **(b)** Uniformly modified particles covered with fluorocarbon chains on the surface; **(c)** JP1s mostly covered by fluorocarbon chains; **(d)** JP2s mostly covered by hydrocarbon chains.

On the bare SiO_2_ particles (Figure 3a), vibrational absorption peaks of O-H are at 3,459 and 1,640 cm^−1^. The intensity of these two absorption peaks on the modified particles decreased or even disappeared. This is due to the condensation reaction between the Si-OH on the bare SiO_2_ particle surface and the silane coupling agent, and the density of hydrogen bonds between the particle surface and water is also reduced. The absorption peaks at 2,949 and 2,850 cm^−1^ are related to the C-H stretching vibration of the methyl group and the methylene group, respectively. The absorption peaks between 1,170 and 1,286 cm^−1^ are the C-F symmetrical deformation of saturated fluorocarbon. And from Figures 3b–d, the intensity of these absorption peaks weakened, indicating that the number of -CF_3_ and -CF_2_ decreased. In summary, it can be preliminarily explained that the modified SiO_2_ particles were covered by different proportions of hydrocarbon and fluorocarbon. Among them, the amount of fluorocarbon on the uniformly modified particle was the largest, followed by JP1, and JP2 was the least.

**Figure 4:**
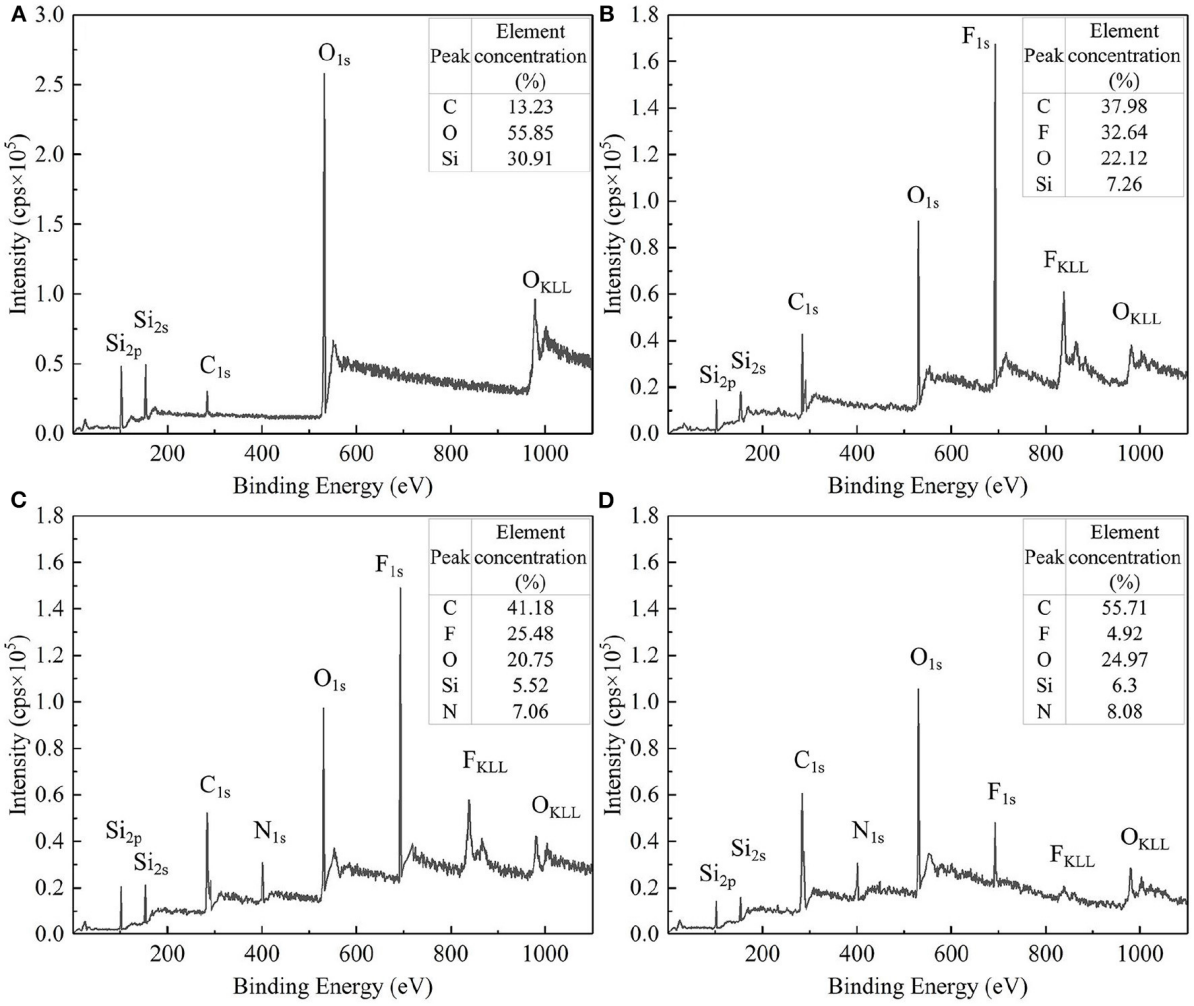
XPS full spectrum and relative element concentration. **(A)** Bare silica particles; **(B)** Uniformly modified particles; **(C)** JP1s; **(D)** JP2s.

X-ray photoelectron spectroscopy (XPS) was used to measure the surface element composition and concentration of bare silica particles and modified particles to further discuss the effectiveness of the modification. For the sake of comparison, the XPS measurement was also performed on the bare silica particles. These test results are illustrated in Figure 4. Compared with bare silica particles (Figure 4A), F_1s_ signal peaks appeared on the surface of modified SiO_2_ particles (Figures 4B–D), indicating that the surface of SiO_2_ particles was effectively grafted with perfluoroalkyl segment. In addition, the relative element concentrations of fluorine on the uniformly modified particles, JP1s and JP2s were detected to decrease sequentially, which proved that the coverage of the perfluoroalkyl segment on the uniformly modified particles, JP1s and JP2s was decreased sequentially.

### Asymmetric Structure of Fluorescent Particles

**Figure 5:**
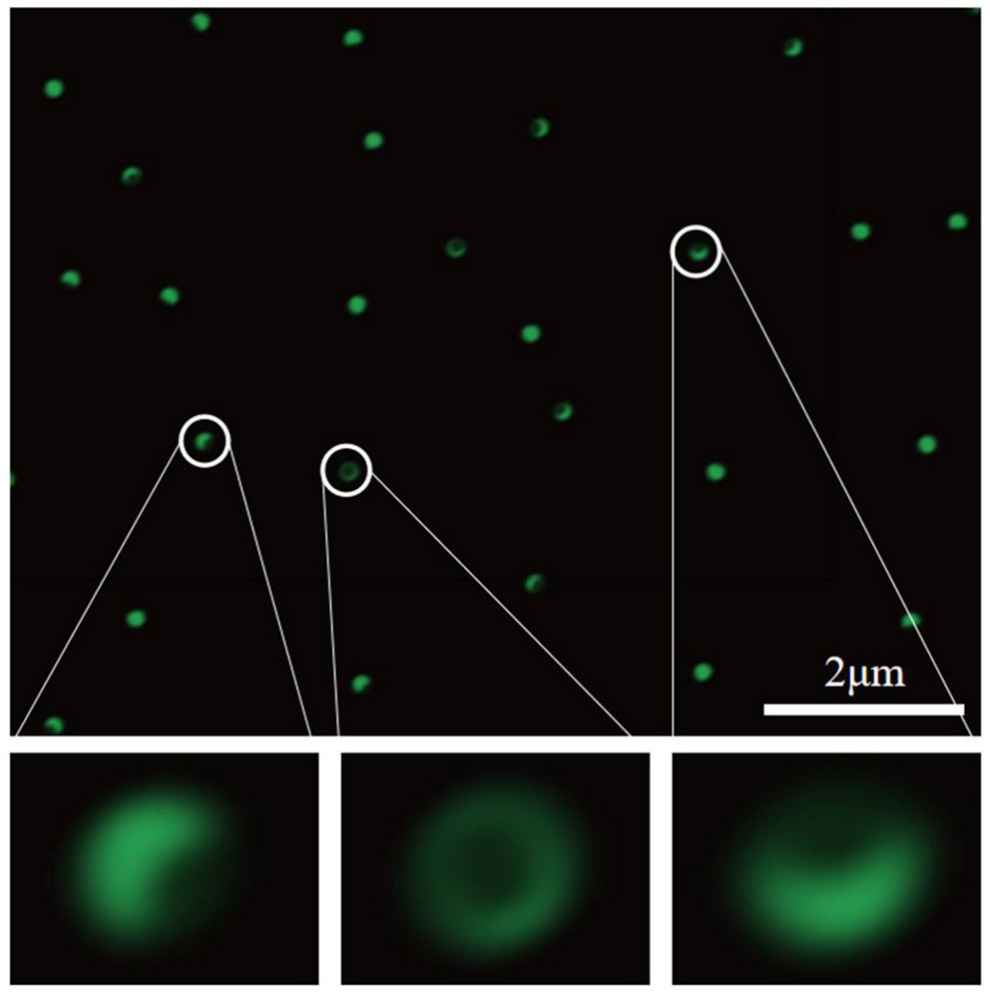
Fluorescence microscope image of Janus particles with green fluorescence dispersed in the solution.

In order to verify that the surface of the synthesized Janus particles has an asymmetric structure, FITC was grafted onto the surface of the APS-coated particles. As shown in Figure 5, one side of the particle surface is significantly stronger in fluorescence than the other side, and the area occupied by the fluorescent portion is larger than that of the non-fluorescent portion. This not only confirms that the prepared Janus particles have an asymmetric structure, but also shows that the two modified regions of the asymmetric structure have different areas. The weak fluorescence on the Janus particle surface grafted with OTS is mainly caused by diffuse reflection and scattering caused by the bright light emitted by the fluorescent group.

**Figure 6:**
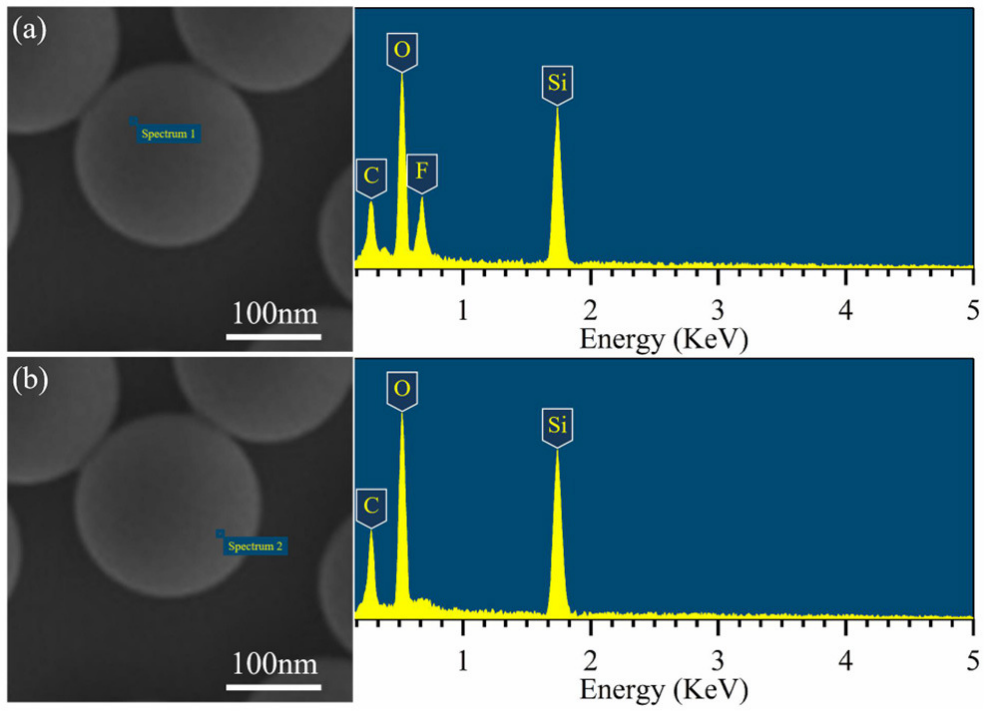
EDS-point scanning of the JP1s. **(a)** Corresponding to the side covered by perfluoroalkyl segments side; **(b)** corresponding to the side covered by alkyl segments.

EDS-point scanning was performed on the JP1s to prove that the functional groups on the synthetic Janus particles have compartmental location differences. Two representative locations of the EDX are shown in Figure 6. In Figure 6a, a significant F element signal peak appears, indicating that the F element was enriched near the scanning point. In the scan point range in Figure 6b, there is no significant F element detected. According to the EDS test principles, there are micro-local differences in the distribution of F element on the JP1, which proves the compartmental distribution of perfluoroalkyl segments and alkyl segments on the synthesized Janus particles.

### Colloidal and Interfacial Properties of Janus Particles

#### Contact Angle and Wettability

**Figure 7:**
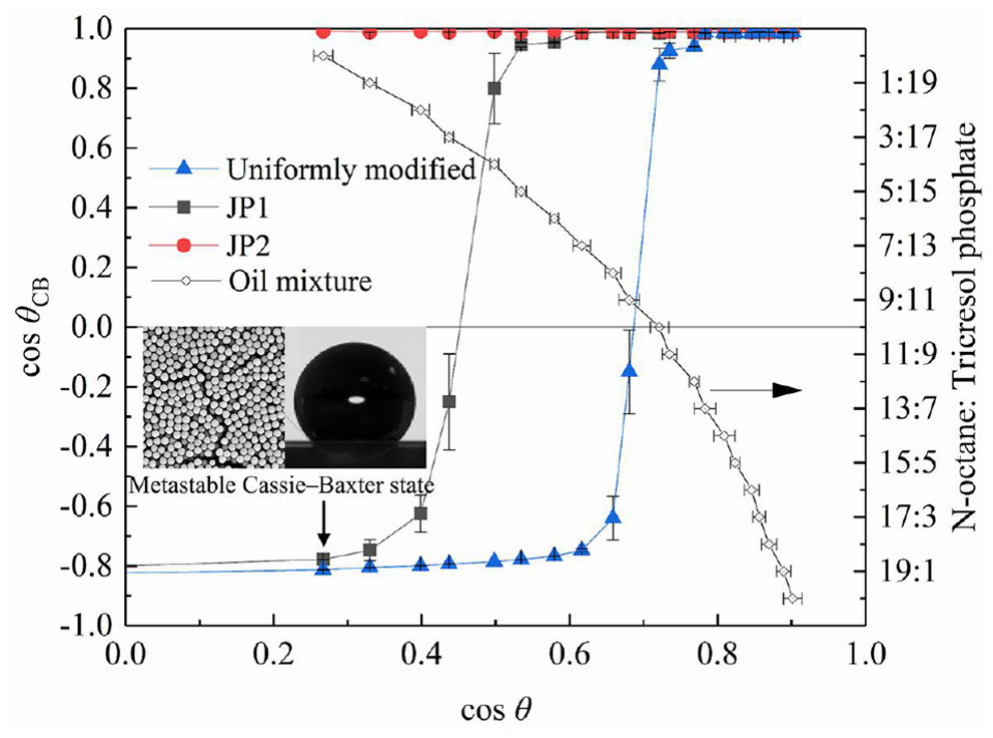
Contact angle of particles. The contact angle data measured on the smooth quartz surface covered by fluorocarbon chains is expressed as cosθ, and the rough contact angle data measured on the quartz slide spin-coated with particles is expressed as cosθ_CB_.

The contact angle measurement results of Figure 7 show that the difference between the rough surface composed of particles and the smooth particle surface is significant. The particles were placed on the substrate by the spin coating method to form a rough surface with closely packed particles (Mihi et al., 2006), so that the measured contact angle deviates from the intrinsic contact angle of particles. The measurement results of the contact angle are consistent with the composite surface described by the Cassie–Baxter equation (Marmur, 2008), that is, a microstructured surface with a concave curvature. In addition, when the contact angle of the rough surface changes significantly (cosθ_CB_ crosses 0 points), the corresponding cosθ value is related to the surface material of the particles. The larger the area of particles covered by fluorocarbon, the more the particles can show oil repellency (cosθ_CB_ > 0) to liquids with lower surface tension (i.e., larger cosθ).

**Figure 8:**
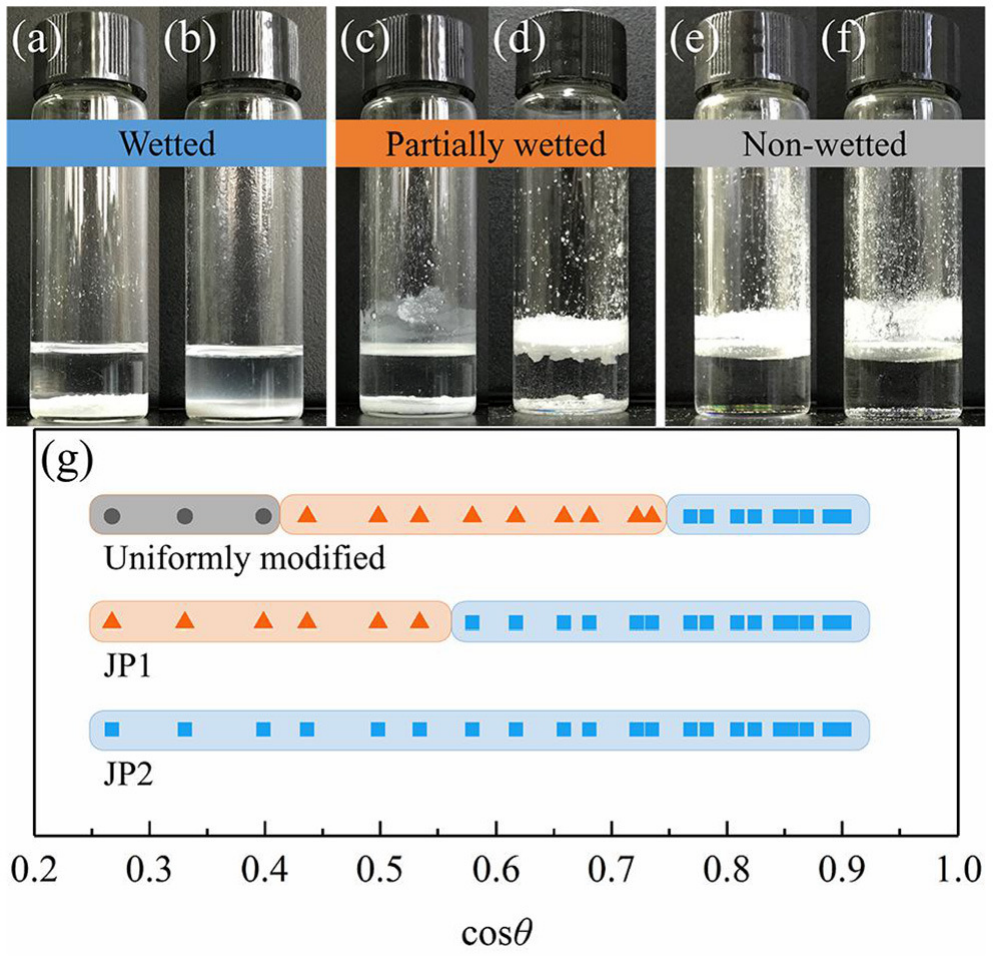
Wettability transition of particles. JP2s were added to **(a)** N-octane: Tricresol phosphate = 3:17 and **(b)** Tricresol phosphate. JP1s were added to **(c)** N-octane: Tricresol phosphate = 3:17 and **(d)** Tricresol phosphate. Uniformly modified particles were added to **(e)** N-octane: Tricresol phosphate = 3:17 and **(f)** Tricresol phosphate. **(g)** Wettability of particles on the oil-air surface measured by adjusting the oil phase substance. Wetted (blue band): particles completely immersed in the oil; Partly wetted (red band): some particles immersed in the oil; Non-wetted (gray band): particles all stay above the oil-air surface.

The wetted state of the particles placed on the surface of all selected stationary oils is given in Figure 8. As the surface tension of the oil increases, the cosθ values obtained from the smooth surface increases, and visibly non-wetted particles begin to appear on the oil surface. The occurrence of such non-wetting particles is correlated with the contact angle results measured from the rough surface (Figure 7). The dry particles placed directly on the oil surface in a fluffy state were not wetted due to the self-assembly of a sufficiently robustness metastable Cassie–Baxter state (Poetes et al., 2010) by particles on oil surface, that is, to form an oil-repellent surface and prevent other particles from contacting the oil phase. In contrast, when the metastable Cassie–Baxter state is not robust enough, the particles or aggregates of particles are immersed in the oil under gentle hand shaking or gravity. Cassie–Baxter state robustness is closely related to particle surface materials. The uniformly modified particles showed non-wetted state in more oil mixtures than JP1s. And JP2s have no wetting inflection point. This is due to the difference in the degree of fluorocarbon coverage. The degree of surface coverage of JP2, JP1, and uniformly modified particle by fluorocarbon chains increases sequentially. Although JP2 is grafted with fluorocarbon, the area covered by the hydrocarbon chain is too large, so that the particles as a whole exhibit strong lipophilicity.

#### Foaming Ability and Surface Tension

**Figure 9:**
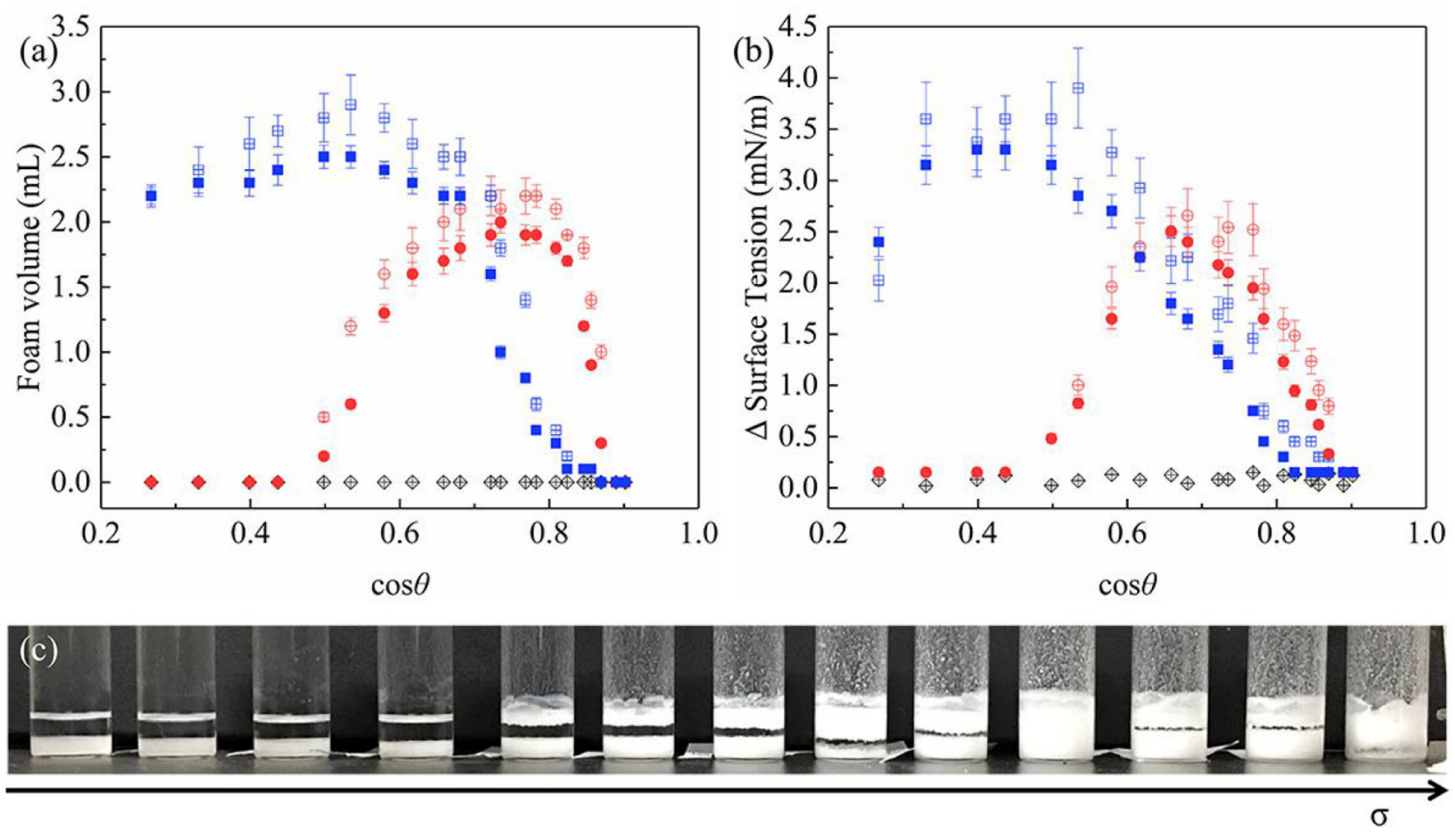
Particle-stabilized oil foam volume and corresponding surface tension difference prepared by hand shaking. **(a)** Foaming volume; **(b)** Oil-air surface tension difference between the original and particle-containing oil; **(c)** An image of JP1 foam systems prepared from a series of oils with gradually increasing surface tension σ (decreasing cosθ). Some foam systems. Round: uniformly modified particles; square: JP1s; diamond: JP2s. Filled and open symbols show the oils containing particles were ultrasonically pre-treated and non-pre-treated, respectively.

As can be seen from Figures 9a,c, JP1s and the uniformly modified particles stabilize the foams, while JP2s wetted in all oil mixture does not participate in foam stabilized. Combined with the experimental results of the oil-air surface tension difference (Figure 9b), the change in foam volume is significantly related to particle surface activity. Due to the asymmetric structure of Janus particles, the maximum surface activity of JP1 is higher than that of uniformly modified particles, which lead to the maximum foam volume of JP1 larger than that of uniformly modified particles. But the foam volume of Janus particles is not always larger than that of uniformly modified particles. Because JP1 and JP2 are more oleophilic than homogeneous particles, Janus particles need to produce foam in liquids with higher surface tension.

The difference in surface tension with and without particles in the oil is shown in Figure 9b. Adsorption of particles on the surface causes a reduction in surface tension. The Δ surface tension and foaming volume of uniformly modified particles and JP1 both have extreme values. When the extremum occurs, cosθ is >0.3 (θ < 72.5°). Referring to Figures 7, 9b, it can be found that the cosθ value corresponding to the maximum value of Δ surface tension is close to the cosθ value at the point where cosθ_CB_ passes through the zero (the lipophilic/oil repellent state of the rough surface changes). This indicates that the adsorption of particles to the surface is directly related to the contact angle θ_CB_ of the rough surface composed of particles, rather than the intrinsic contact angle θ of the surface of a single particle. In order to further prove this inference, the size of particle agglomerates was measured.

#### Effects of Particle Agglomerates

**Figure 10:**
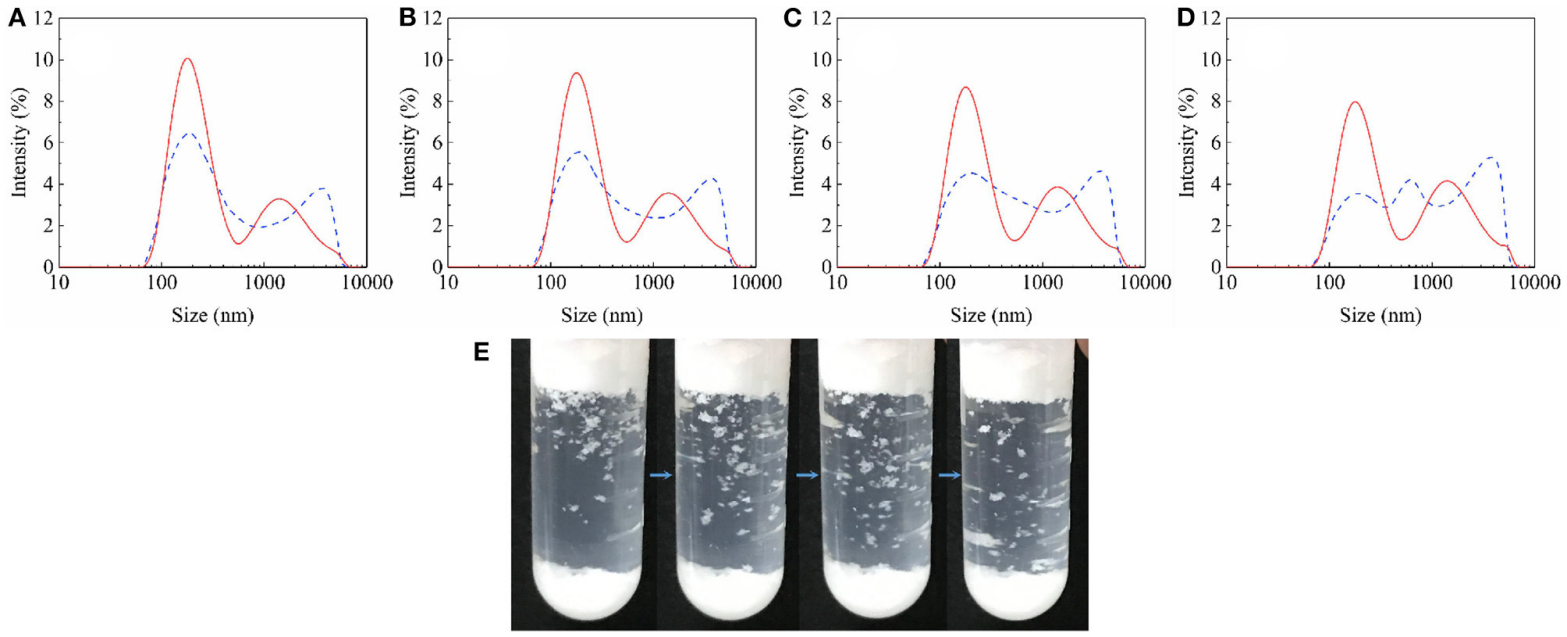
Presence of JP1 agglomerates in oil mixture. Solid line: ultrasonic pretreated; Dashed line: unpretreated. **(A)** N-octane: Tricresol phosphate = 7:13; **(B)** N-octane: Tricresol phosphate = 5:15; **(C)** N-octane: Tricresol phosphate = 3:17; **(D)** N-octane: Tricresol phosphate = 1:19; **(E)** After the ultrasonic pretreated particle-containing oil (N-octane: Tricresol phosphate = 3:17) is foamed by hand shaking, flocculated aggregates visible to the naked eye fall off from top.

It is worth noting that when the particles were first dispersed in the oil with ultrasonic waves and then shaken in the same way, the particle-stabilized foam volume decreased (Figure 9a). In this regard, we compared the size of agglomerates in oil with and without ultrasonic pretreatment. The results of using a dynamic light scattering technique to represent the diameter of particle agglomerates in a liquid are shown in Figure 10. The dotted lines in Figures 10a–d indicate the presence of particle agglomerates in the series of solutions. In the solutions without ultrasonic pretreatment, the average diameter of the widely distributed substance is significantly larger than that of a single particle (200 nm). This confirms the presence of significant particle agglomerates in the clear solutions. Generally, due to the huge surface energy, most particles have agglomerated spontaneously in the air and liquids. A small amount of air is sealed inside the agglomerates of particles that are immersed in the oil phase provides additional pressure to prevent further penetration. Such particle agglomerates also forms a composite surface described by the Cassie–Baxter equation, which protects the particles constituting the agglomerates from being completely wetted, and makes agglomerates have a reasonable oil repellency (the value of cosθ_CB_ is close to 0), thereby facilitating the adsorption of the agglomerates thermodynamically (Ondarçuhu et al., 1990; Binks and Fletcher, 2001). The data of Δ surface tension with ultrasonic pretreatment (Figure 9b) also indicate that after agglomerates broken up, the wetted particles reduce the tendency to adsorb to the surface.

However, foams were still formed after the action of ultrasound (Figure 9a). After vigorous shaking by hand, whether the particles have been ultrasonic pretreated, they have the opportunity to re-form larger, visible aggregates (Figure 10E). In addition, due to the large number of particles, the particle agglomerates became very large and obvious precipitation occurred. Ultrasound cannot completely disperse these precipitates. Even if the agglomerates in the precipitate are dispersed into smaller states under the action of ultrasound, there are still existing particle agglomerates with Cassie–Baxter state and eventually precipitated again.

**Figure 11:**
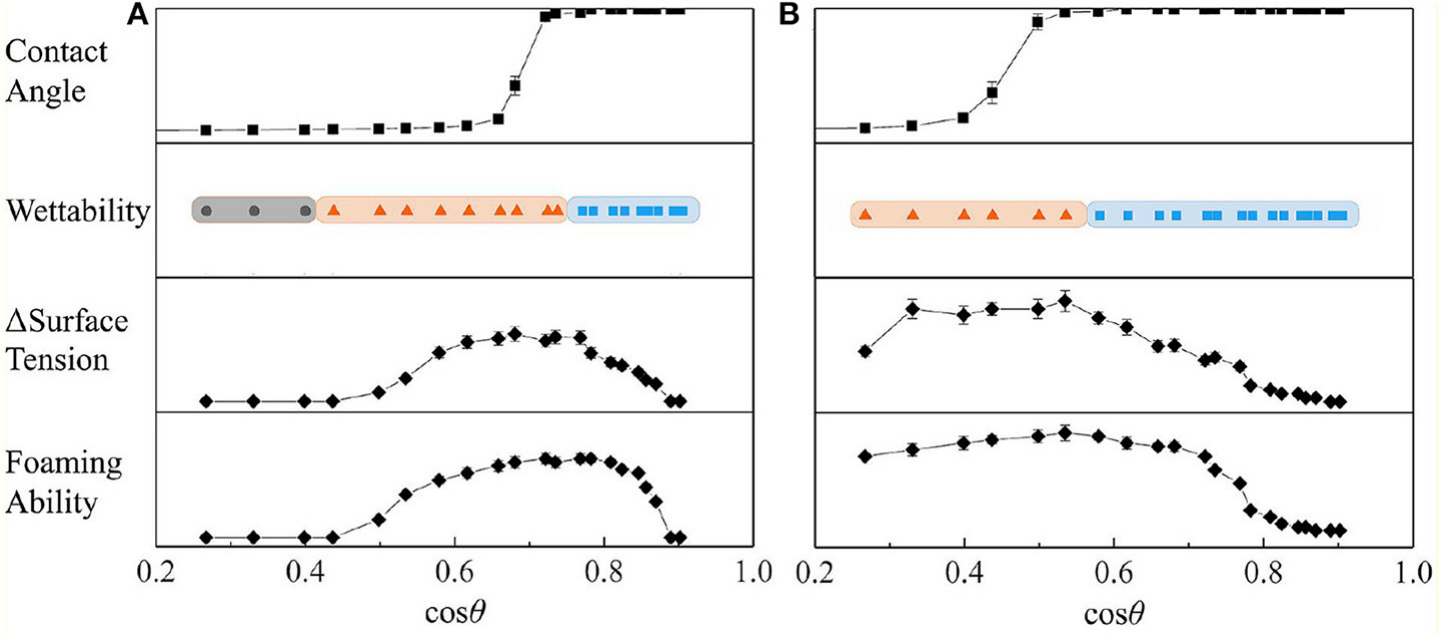
Particles are mainly adsorbed to the surface in an aggregate state. The particle agglomerates with Cassie–Baxter composite surface have proper contact angle and wettability, which leads to the surface tension reduction and foaming ability of particles. Individual particles do not have a proper contact angle, which is not easy to be adsorbed to the surface. **(A)** Uniformly modified particle; **(B)** JP1.

In summary, the particles are not only adsorbed from the bulk phase to the surface in the form of a single particle, but also move toward the surface in an aggregated state. Whether the particles can reach the surface depends mainly on the wettability of the agglomerates. When the contact angle of the particle agglomerates is close to 90°, the particles can be effectively adsorbed on the oil-air surface. However, when the intrinsic contact angle of the surface of a single particle approaches 90°, the agglomerates do not tend to adsorb to the surface. We summarize the correlations associated with particle agglomeration and surface adsorption in Figure 11.

## Conclusions

1. Using the same Pickering emulsion, two types of Janus particle with different surface modification degrees were synthesized and used for the preparation of oil foams.
2. Influenced by the contact angle of the particle surface, the surface activity of Janus particles is not always greater than that of uniformly modified particles, which is reflected in the ability to reduce surface tension and the volume of foam generated.
3. The particles of JP1 are not only adsorbed from the bulk phase to the surface in the form of a single particle, but also move toward the surface in an aggregated state. For particles that suitable for adsorbing on the oil surface and increasing the foaming volume, the contact angle of the rough surface of particle agglomerates is about 90°, while the inherent contact angle of a single particle is <90°.

## Data Availability Statement

The raw data supporting the conclusions of this article will be made available by the authors, without undue reservation.

## Author Contributions

KW conceived and designed the experiments. GL and CL conducted experiments, recorded data, and analyzed the data. KW and GL wrote the paper.

## Conflict of Interest

The authors declare that the research was conducted in the absence of any commercial or financial relationships that could be construed as a potential conflict of interest.

## References

[B1] Ahmed Z. Mohammad S. Mohammad C. Hosseini-Nasab M. (2020). Foam EOR performance in homogeneous porous media: simulation versus experiments. J. Pet. Explor. Prod. Technol. 10, 2045–2054. 10.1007/s13202-020-00845-0

[B2] Alargova R. G. Warhadpande D. S. Paunov V. N. Velev O. D. (2004). Foam superstabilization by polymer microrods. Langmuir 20, 10371–10374. 10.1021/la048647a 15544360

[B3] Asghari A. K. Norton I. Mills T. Sadd P. Spyropoulos F. (2016). Interfacial and foaming characterisation of mixed protein-starch particle systems for food-foam applications. Food Hydrocoll. 53, 311–319. 10.1016/j.foodhyd.2015.09.007

[B4] Aveyard R. (2012). Can Janus particles give thermodynamically stable pickering emulsions? Soft Matter 8, 5233–5240. 10.1039/c2sm07230k

[B5] Binks B. Davies C. Fletcher P. Sharp E. (2010). Non-aqueous foams in lubricating oil systems. Colloids Surf. A Physicochem. Eng. Asp. 360, 198–204. 10.1016/j.colsurfa.2010.02.028

[B6] Binks B. P. Fletcher P. (2001). Particles adsorbed at the oil– water interface: a theoretical comparison between spheres of uniform wettability and “Janus” particles. Langmuir 17, 4708–4710. 10.1021/la0103315

[B7] Binks B. P. Horozov T. S. (2005). Aqueous foams stabilized solely by silica nanoparticles. Angew. Chem. Int. Edn. 44, 3722–3725. 10.1002/anie.200462470 15887197

[B8] Binks B. P. Rocher A. (2010). Stabilisation of liquid–air surfaces by particles of low surface energy. Phys. Chem. Chem. Phys. 12, 9169–9171. 10.1039/c0cp00777c 20571705

[B9] Binks B. P. Rocher A. Kirkland M. (2011). Oil foams stabilised solely by particles. Soft Matter 7, 1800–1808. 10.1039/C0SM01129K15887197

[B10] Binks B. P. Tyowua A. T. (2013). Influence of the degree of fluorination on the behaviour of silica particles at air–oil surfaces. Soft Matter 9, 834–845. 10.1039/C2SM27395K

[B11] Bournival G. Ata S. Wanless E. J. (2015). The roles of particles in multiphase processes: Particles on bubble surfaces. Adv. Colloid Interface Sci. 225, 114–133. 10.1016/j.cis.2015.08.008 26344866

[B12] Cassie A. Baxter S. (1944). Wettability of porous surfaces. Trans. Faraday Soc. 40, 546–551. 10.1039/tf9444000546

[B13] Davarpanah A. Akbari E. Doudman-Kushki M. Ketabi H. Hemmati M. (2019). Simultaneous feasible injectivity of 2019 foam and hydrolyzed polyacrylamide to optimize the oil recovery enhancement. Energy Explor. Exploit. 37, 44–59. 10.1177/0144598718786022

[B14] De Gennes P.-G. (1992). Soft matter. Rev. Mod. Phys. 64:645 10.1103/RevModPhys.64.645

[B15] Dickinson E. Ettelaie R. Kostakis T. Murray B. S. (2004). Factors controlling the formation and stability of air bubbles stabilized by partially hydrophobic silica nanoparticles. Langmuir 20, 8517–8525. 10.1021/la048913k 15379469

[B16] Domingues E. M. Arunachalam S. Nauruzbayeva J. Mishra H. (2018). Biomimetic coating-free surfaces for long-term entrapment of air under wetting liquids. Nat. Commun. 9:3606. 10.1038/s41467-018-05895-x 30190456 PMC6127334

[B17] Emadi S. Shadizadeh S. R. Manshad A. K. Rahimi A. M. Nowrouzi I. Mohammadi A. H. (2019). Effect of using Zyziphus spina christi or Cedr Extract (CE) as a natural surfactant on oil mobility control by foam flooding. J. Mol. Liq. 293:111573 10.1016/j.molliq.2019.111573

[B18] Fox H. Zisman W. (1950). The spreading of liquids on low energy surfaces. I. polytetrafluoroethylene. J. Colloid Sci. 5, 514–531. 10.1016/0095-8522(50)90044-4

[B19] Georgieva D. Cagna A. Langevin D. (2009). Link between surface elasticity and foam stability. Soft Matter 5, 2063–2071. 10.1039/b822568k

[B20] Glaser N. Adams D. J. Böker A. Krausch G. (2006). Janus particles at liquid–liquid interfaces. Langmuir 22, 5227–5229. 10.1021/la060693i 16732643

[B21] Hong L. Jiang S. Granick S. (2006). Simple method to produce janus colloidal particles in large quantity. Langmuir 22, 9495–9499. 10.1021/la062716z 17073470

[B22] Horozov T. S. (2008). Foams and foam films stabilised by solid particles. Curr. Opin. Colloid Interface Sci. 13, 134–140. 10.1016/j.cocis.2007.11.009

[B23] Hunter T. N. Pugh R. J. Franks G. V. Jameson G. J. (2008). The role of particles in stabilising foams and emulsions. Adv. Colloid Interface Sci. 137, 57–81. 10.1016/j.cis.2007.07.007 17904510

[B24] Hussain A. A. A. Vincent-Bonnieu S. Bahrim R. Z. K. Pilus R. M. Rossen W. R. (2019). The impacts of solubilized and dispersed crude oil on foam in a porous medium. Colloids Surf. A Physicochem. Eng. Asp. 579:123671 10.1016/j.colsurfa.2019.123671

[B25] Kaptay G. (2006). On the equation of the maximum capillary pressure induced by solid particles to stabilize emulsions and foams and on the emulsion stability diagrams. Colloids Surf. A Physicochem. Asp. 282, 387–401. 10.1016/j.colsurfa.2005.12.021

[B26] Marmur A. (2008). From hygrophilic to superhygrophobic: theoretical conditions for making high-contact-angle surfaces from low-contact-angle materials. Langmuir 24, 7573–7579. 10.1021/la800304r 18543997

[B27] Mihi A. Ocaña M. Míguez H. (2006). Oriented colloidal-crystal thin films by spin-coating microspheres dispersed in volatile media. Advanced Materials. 18, 2244–2249. 10.1002/adma.200600555

[B28] Murakami R. Bismarck A. (2010). Particle-stabilized materials: dry oils and (polymerized) non-aqueous foams. Adv. Funct. Mater. 20, 732–737. 10.1002/adfm.200902007

[B29] Murray B. S. Ettelaie R. (2004). Foam stability: proteins and nanoparticles. Curr. Opin. Colloid Interface Sci. 9, 314–320. 10.1016/j.cocis.2004.09.004

[B30] Narsimhan G. (2016). Drainage of particle stabilized foam film. Colloids Surf. A Physicochem. Eng. Asp. 495, 20–29. 10.1016/j.colsurfa.2016.01.044

[B31] Ondarçuhu T. Fabre P. Raphaël E. Veyssié M. (1990). Specific properties of amphiphilic particles at fluid interfaces. J. Phys. 51, 1527–1536. 10.1051/jphys:0199000510140152700

[B32] Poetes R. Holtzmann K. Franze K. Steiner U. (2010). Metastable underwater superhydrophobicity. Phys. Rev. Lett. 105:166104. 10.1103/PhysRevLett.105.166104 21230986

[B33] Pugh R. (1996). Foaming, foam films, antifoaming and defoaming. Adv. Colloid Interface Sci. 64, 67–142. 10.1016/0001-8686(95)00280-4

[B34] Sakthivel S. Adebayo A. Kanj M. Y. (2019). Experimental evaluation of carbon dots stabilized foam for enhanced oil recovery. Energy Fuels 33, 9629–9643. 10.1021/acs.energyfuels.9b02235

[B35] Sheludko A. (1967). Thin liquid films. Adv. Colloid Interface Sci. 1, 391–464. 10.1016/0001-8686(67)85001-2

[B36] Shrestha L. K. Aramaki K. Kato H. Takase Y. Kunieda H. (2006). Foaming properties of monoglycerol fatty acid esters in nonpolar oil systems. Langmuir 22, 8337–8345. 10.1021/la061204h 16981746

[B37] Shrestha L. K. Shrestha R. G. Sharma S. C. Aramaki K. (2008). Stabilization of nonaqueous foam with lamellar liquid crystal particles in diglycerol monolaurate/olive oil system. J. Colloid Interface Sci. 328, 172–179. 10.1016/j.jcis.2008.08.051 18823901

[B38] Shrestha R. G. Shrestha L. K. Solans C. Gonzalez C. Aramaki K. (2010). Nonaqueous foam with outstanding stability in diglycerol monomyristate/olive oil system. Colloids Surf. A Physicochem. Eng. Asp. 353, 157–165. 10.1016/j.colsurfa.2009.11.007

[B39] Singh R. Mohanty K. K. (2020). Study of nanoparticle-stabilized foams in harsh reservoir conditions. Transp. Porous Media 131, 135–155. 10.1007/s11242-018-1215-y

[B40] Song M. Ju J. Luo S. Han Y. Dong Z. Wang Y. (2017). Controlling liquid splash on superhydrophobic surfaces by a vesicle surfactant. Sci. Adv. 3:e1602188 10.1126/sciadv.1602188 28275735 PMC5332151

[B41] Sun Q. Li Z. Wang J. Li S. Li B. Jiang L. (2015). Aqueous foam stabilized by partially hydrophobic nanoparticles in the presence of surfactant. Colloids Surf. A Physicochem. Eng. Asp. 471, 54–64. 10.1016/j.colsurfa.2015.02.007

[B42] Wang J. Nguyen A. (2016). Foam drainage in the presence of solid particles. Soft Matter 12, 3004–3012. 10.1039/C6SM00028B 26877265

[B43] Yan Y. Y. Gao N. Barthlott W. (2011). Mimicking natural superhydrophobic surfaces and grasping the wetting process: a review on recent progress in preparing superhydrophobic surfaces. Adv. Colloid Interface Sci. 169, 80–105. 10.1016/j.cis.2011.08.005 21974918

